# Potential SARS-CoV-2 RdRp inhibitors of cytidine derivatives: Molecular docking, molecular dynamic simulations, ADMET, and POM analyses for the identification of pharmacophore sites

**DOI:** 10.1371/journal.pone.0273256

**Published:** 2022-11-28

**Authors:** Sarkar M. A. Kawsar, Mohammed A. Hosen, Sajjad Ahmad, Youness El Bakri, Hamid Laaroussi, Taibi Ben Hadda, Faisal A. Almalki, Yasuhiro Ozeki, Souraya Goumri-Said

**Affiliations:** 1 Faculty of Science, Department of Chemistry, Laboratory of Carbohydrate and Nucleoside Chemistry (LCNC), University of Chittagong, Chittagong, Bangladesh; 2 Department of Health and Biological Sciences, Abasyn University, Peshawar, Pakistan; 3 Department of Theoretical and Applied Chemistry, South Ural State University, Chelyabinsk, Russian Federation; 4 Faculty of Sciences, Laboratory of Applied Chemistry & Environment, Mohammed Premier University, Oujda, Morocco; 5 Faculty of Pharmacy, Department of Pharmaceutical Chemistry, Umm AlQura University, Makkah, Saudi Arabia; 6 School of Sciences, Yokohama City University, Yokohama, Japan; 7 Physics Department, College of Science, Alfaisal University, Riyadh, Saudi Arabia; Universiti Teknologi Malaysia, MALAYSIA

## Abstract

The RNA-dependent RNA polymerase (RdRp) of SARS-CoV-2 is one of the optimum targets for antiviral drug design and development. The hydroxyl groups of cytidine structures were modified with different aliphatic and aromatic groups to obtain 5´-*O*-acyl and 2´,3´-di-*O*-acyl derivatives, and then, these derivatives were employed in molecular modeling, antiviral prediction, molecular docking, molecular dynamics, pharmacological and POM studies. Density functional theory (DFT) at the B3LYP/6-31G++ level analyzed biochemical behavior and molecular electrostatic potential (MESP) of the modified cytidine derivatives. The antiviral parameters of the mutated derivatives revealed promising drug properties compared with those of standard antiviral drugs. Molecular docking has determined binding affinities and interactions between the cytidine derivatives and SARS-CoV-2 RdRp. The modified derivatives strongly interacted with prime Pro620 and Lys621 residues. The binding conformation and interactions stability were investigated by 200 ns of molecular dynamics simulations and predicted the compounds to firmly dock inside the RdRp binding pocket. Interestingly, the binding residues of the derivatives were revealed in high equilibrium showing an enhanced binding affinity for the molecules. Intermolecular interactions are dominated by both Van der Waals and electrostatic energies. Finally, the pharmacokinetic characterization of the optimized inhibitors confirmed the safety of derivatives due to their improved kinetic properties. The selected cytidine derivatives can be suggested as potential inhibitors against SARS-CoV-2. The POM Theory supports the hypothesis above by confirming the existence of an antiviral (O^δ-^—O’^δ-^) pharmacophore site of Hits.

## Introduction

Nucleoside agents (NAs) are the subunits of DNA and RNA and comprise a sugar moiety connected to a nitrogen base through an *N*-β-glycosidic bond [1]. NAs have considerable clinical importance as medicinal agents due to their antiviral and anticancer activities [2] and are the drugs of choice for treating various viral diseases, such as herpes simplex (HSV-1), human cytomegalovirus, varicella-zoster, human immunodeficiency virus (HIV) type-1, human hepatitis B (HBV) and C (HCV) [3], ebola [4], dengue [5], and Zika [6]. Additionally, 2ʹ-deoxynucleosides such as idoxuridine, trifluridine, vidarabine, and brivudine are used to treat herpes virus infection [7, 8]. Certain 2ʹ,3ʹ-dideoxynucleosides such as zidovudine, didanosine, zalcitabine, stavudine, and abacavir are the most effective therapeutic agents against HIV [9]. Modifications in sugar moieties, such as ribofuranose or deoxyribofuranose of nucleosides, include changes in sugar substituents, the substitute of oxygen with another atom, and the inclusion of a heteroatom in the sugar ring, ring size variations, and replacement with an acyclic moieties [10–15]. These alterations may lead to excellent variations in the biological activity and degree of selective toxicity according to the respective chemical and physical properties of the moieties [16–21]. The modified compounds exhibit a broad-spectrum biological activity. For example, zidovudine with an azido group at 3ʹ-position is used to treat HIV. Thymidine (**1**) derivatives such as telbivudine are antiviral drugs are used in HBV treatment [22]. Azidothymidine (3ʹ-azido-2ʹ,3ʹ-dideoxythymidine) is another thymidine analog used in HIV treatment. The supplementation of dietary cytidine (5´)-diphosphocholine protects against the development of memory deficits [23]. Cytidine is present in organ meats and pyrimidine-rich foods such as beer, tomatoes, broccoli, and oats. Cytidine is an RNA component that transfers instructions from DNA to protein [24]. When RNA levels decrease, cytidine is supplemented to maintain high RNA levels for a high memory function. Another important function of cytidine is to increase dopamine production and release it in the brain. Cytidine is a powerful neurotransmitter responsible for regulating functions, such as mood and movement control.

Nucleoside analogs and nucleobases constitute a pharmacologically diverse family, which includes cytotoxic compounds, antiviral agents, and immune suppressive molecules [15, 21, 25–27]. Cytidine analog 5-AZA-2^´^-deoxycytidine is utilized to control the growth of neuroblastoma malignant tumor [28]. Cytidine analog KP-1461 is an anti-HIV agent that acts as a viral mutagen [29]. Various cytidine derivatives modified at the base or ribose exhibit antiviral or antitumor activities.

The recent outbreak of the novel coronavirus disease (COVID-19), caused by a severe acute respiratory syndrome (SARS)-like coronavirus, that started in Wuhan, China, is spreading rapidly in humans; this outbreak is now considered a global pandemic [30]. Modifications of the hydroxyl (–OH) group of the nucleoside structure illustrated some potent SARS-CoV-2 candidates [31, 32] and antimicrobial agents. The COVID-19 outbreak caused by the new coronavirus, which appeared in China, remains a serious problem worldwide. Although SARS-CoV and SARS-CoV-2 agents belong to a beta-coronavirus category, they slightly differ from each other. Studies have shown that SARS-CoV-2 shares 80% nucleotide identity and 89.10% nucleotide similarity with SARS-CoV. Thus, the RNA-dependent RNA polymerase of SARS-CoV, RdRp, is the target of several *in silico* investigations for developing potential inhibitor candidates. Between nCoV and nCoV2, RdRp provides a high sequence identity rate; hence, their RdRp is likely homologous and has similar structure and functions. Furthermore, SARS-CoV and SARS-CoV-2 affect cells in the same manner and employ the same protein machinery to enhance inside the host cell. The interaction results of FDA drugs with the apo form of COVID-19 M^pro^ and spike glycoprotein can play an important role in the treatment of COVID-19 [33]. Luteolin (the main flavonoid in honeysuckle) was found to bind with a high affinity to the same sites of the main protease of SARS-CoV-2 as the control molecule. Interactions with the main protease may play a key role in fighting against viruses [34–36]. Cepharantine also showed the interactions of apo and holo forms of COVID-19 main protease enzyme (M^pro^) and spike glycoprotein of SAR SCoV-2 [37]. Due to their features, we explored the MESP and biochemical behavior of several previously synthesized cytidine derivatives by conducting a quantum mechanical study. Furthermore, all the derivatives were employed for molecular docking against SARS-CoV-2 RdRp protein (PDB: 6M71) to understand their non-binding interactions, binding mode, and binding affinity and to predict their antiviral properties. The dynamic stability of the high-affinity complexes was checked using all atoms 200 ns molecular dynamics simulation and the docked predictions were validated by the MMGBSA binding free energy method. Pharmacokinetic properties were investigated to compare their absorption, lipophilicity, and solubility, and a radar map was utilized to understand their biological acceptance.

## Computational details

### Methods

To identify drug interactions with receptor proteins, molecular docking is the optimum tool. In the blind docking method, the overall surface of the protein molecule was thoroughly analyzed for binding sites. The following software tools were used in this study to predict antiviral properties: i) Gaussian 09, ii) AutoDock 4.2.6, iii) Swiss-Pdb 4.1.0, iv) Python 3.8.2, v) Discovery Studio 4.1, vi) PyMOL 2.3, vii) http://crdd.osdd.net/servers/avcpred. Moreover, the admetSAR server (http://lmmd.ecust.edu.cn/admetsar2/about), and SwissADME free web tools (http://www.swissadme.ch) were employed to calculate the pharmacokinetic properties.

### Antiviral activity determination

Antiviral molecules (AVMs) present a category of antimicrobial drugs used to treat viral infections by inhibiting the growth of viral pathogens inside the host cells. For antiviral activity calculation, we used online software (http://crdd.osdd.net/servers/avcpred), which showed the inhibitory percentage. The SD (sampled data) file format of the cytidine derivatives was entered as input for predications. The derivatives were assessed for the development of antiviral therapeutics and suggested the optimal inhibitory cytidine derivatives for further studies.

### Chemical reactivity

In a computer-based drug design, thermal, molecular orbital, and molecular electrostatic peculiarities are widely calculated using the quantum mechanical method [38]. The geometrical calculation and subsequent alteration of all the cytidine derivatives were conducted using Gaussian 09 program [39]. The thermodynamic properties of the cytidine derivatives were optimized and calculated employing density functional theory (DFT) with Beck’s (B) three-feature hybrid model and Lee, Yang, and Parr’s correlation functional by using a basis set B3LYP/6-31G++ [40, 41].

### Protein selection and molecular docking

**Fig 1:**
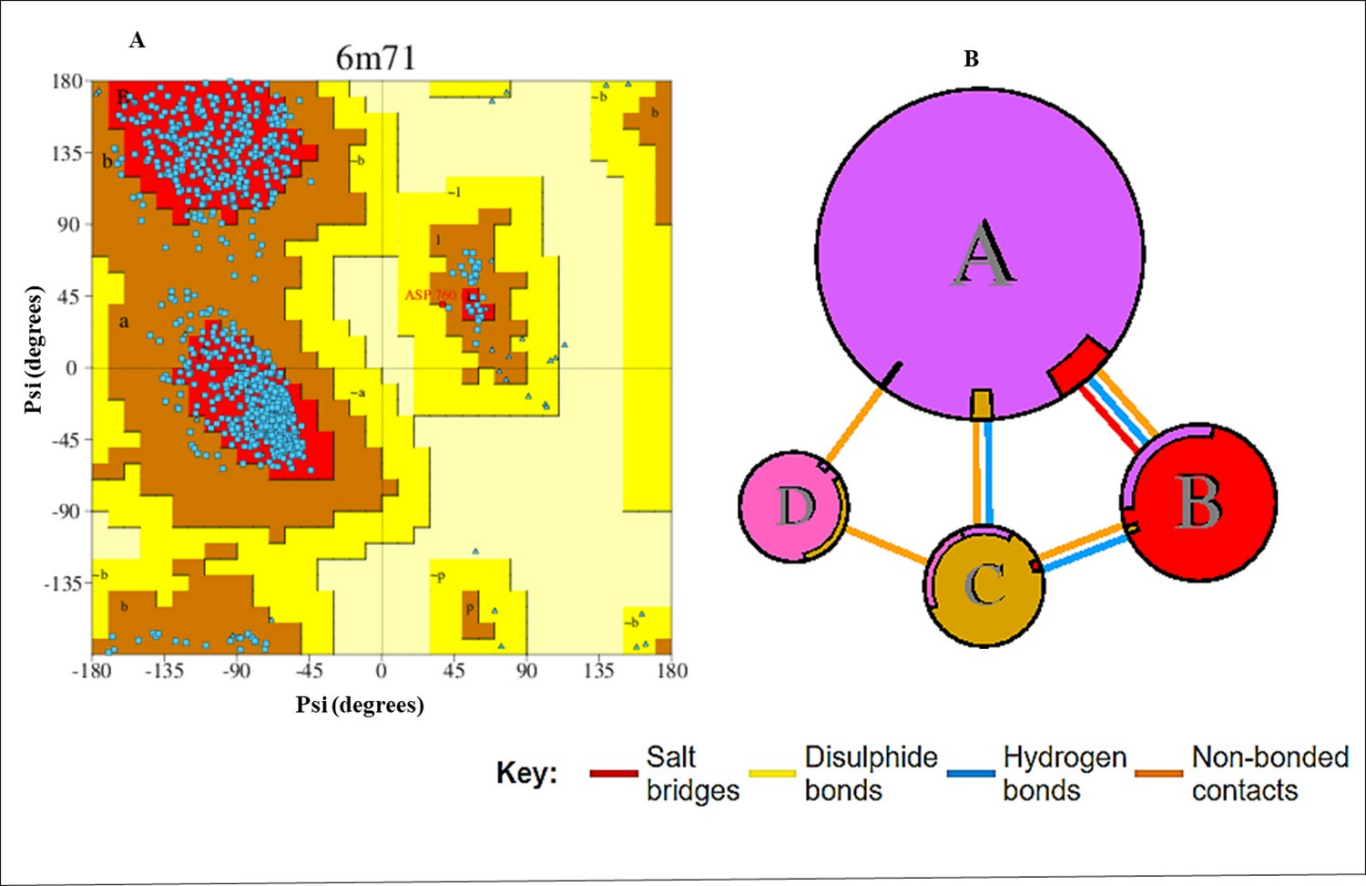
(**A**): Ramachandran plot. SARS-CoV-2 RdRp (pdb: 6M71) protein and (**B**): Interfaces summary of SARS-CoV-2 RdRp (pdb: 6M71).

The 3D crystal structure of SARS-CoV-2 RdRp (pdb: 6M71, 7bv2, and 7B3B) was retrieved from protein data bank [42]. PyMol (version 1.3) software packages were used to eliminate all associated heteroatoms and water molecules [43]. Protein energy was minimized using UCSF Chimera version 1.15 [44]. Furthermore, a molecular docking study was conducted between the enzymes and cytidine derivatives drugs using PyRx version 0.8 [45]. The polar hydrogens and Kollman charges were added to the protein using the AutoDock tool. The grid box size set on the enzyme as 120.6361 Å × 115.6029 Å × 116.6400 Å and 26.0286 Å × 44.5394 Å × 43.1279 Å along the X-, Y-, and Z-axis, respectively [46]. The value of 0.375 Å was set between the grid points. The number of docking runs for each compound is 100 and each iteration is affiliated with AutoDock binding energy in kcal/mol. After docking, the best docked binding mode of compounds was complexed with the receptor enzyme and visualized in Accelrys Discovery Studio version 2021. Validation of the docking protocol involved retrieval of PDB ID: 7B3B, extracting co-crystallized remdesivir, and redocking at the same position using the protocol described above. It was observed that the docking protocol revealed the same intermolecular conformation as reported by the crystal structure, thus pointing to the correct docking procedure. The visualization analysis involves non-binding interactions among the cytidine derivatives and amino acid chains of receptor enzyme [47]. PDBsum online server was also used to check the validation of the SARS-CoV-2 RdRp (pdb: 6M71) receptor with Ramachandran plot and interfaces summary (Fig 1) which revealed that >87.0% residues are in the allowed region and no residues were missed.

### Molecular dynamics simulations

Molecular dynamics simulations of RdRp**—**compound **7**, RdRp-compound **8**, RdRp-compound **9**, RdRp-compound **13,** and RdRp-compound **14** were undertaken using AMBER20 software [48]. Initial preprocessing of the complexes was performed using an antechamber program. The compounds and RdRp enzyme were parameterized using general AMBER force field (GAFF) [49] and ff14SB force field [50], respectively while topologies were recorded via leap module. Each complex was then neutralized and solved into TIP3P water model [51]. The energy of the systems was minimized via 1500 steps of conjugate gradient and steepest gradient methods. The non-bonded interactions were restrained at a distance of 8 Å. Then the systems were heated for 10 ps at constant temperature and volume (canonical ensemble). Afterward, the systems were equilibrated for 100 ps under periodic boundary conditions under a constant Langevin thermostat. Simulations were carried out for 200 ns for each system explicitly in the isothermal-isobaric ensemble. The long-term electrostatic effects were handled using the particle-mesh-Ewald method (PME) [52]. The hydrogen bond length was constrained via SHAKE algorithm [53] while temperature control was achieved through the Langevin algorithm. CPPTRAJ module [54] was employed to structurally analyze simulation trajectories and plots were drawn using XMGRACE. Binding energies of the systems were estimated using MMPBSA.py module [55] using 1000 snapshots from the simulation trajectories.

### Pharmacokinetic prediction

In drug development, ADMET (absorption, distribution, metabolism, excretion, and toxicity) property prediction is crucial to prevent drug failure in clinical stages. Thus, the developed derivatives were assessed for their *in silico* pharmacokinetic parameters to prevent their collapse during clinical trials and iRdRpve their candidacy as potential candidate drugs. Online server admetSAR was employed to calculate the pharmacokinetic properties of the designed cytidine derivatives and parent compounds. We exploited the online database, admetSAR, to evaluate the pharmacokinetics profile involved in the drug lipophilicity, toxicity, and absorption of cytidine and its selected analogs [56]. By using the structural resemblance exploration methodology, admetSAR predicted the latest and most widespread, manually curated results for several chemicals pertained to the studied ADMET profiles. For ADMET calculation, admetSAR was employed, in which 96,000 sole compounds (including 45 types of ADMET-related parameters), proteins, species, and organisms are diligently curated from various studies.

### Pharmacophore sites identification (POM theory)

The POM (Petra/Osiris/Molinspiration) analyses give substantial ideas about the structural features responsible for their combined antibacterial/antifungal activity and provide guidelines for further modifications, to improve each activity and selectivity of designed drugs targeting potentially the drug-resistant microorganisms.

## Results and discussion

The antiviral efficacy of nucleoside analogs was drawn the attention of scientists many years ago. Cytidine, thymidine, and uridine has antiviral activity against, HIV, HSV-1, human cytomegalovirus, HIV type-1, HBV, dengue, and Zika. Recently, nucleoside analogs play a vital role in the development of COVID-19 drug and showed promising activity. Nucleoside moiety can firmly interact with the main protease and inhibit viral replication. Moreover, aliphatic and aromatic derivatives of nucleosides increased the binding affinity. The number of carbon atoms in the aliphatic chain and the presence of heteroatom in aromatic also enhance the antiviral behavior of nucleoside derivatives. In this study, 14 cytidine derivatives were modified with different aliphatic and aromatic chains (**2**–**15**) (Table 1) and were geometrically optimized to realize the modes of their antimicrobial behavior. Initially, partial acylated derivatives were designated for antiviral activities using the online web tool. Subsequently, the observed activities were rationalized by measuring the IR frequency, physicochemical properties, molecular docking, *in silico* pharmacokinetics, and drug-likeness properties. In nucleoside chemistry, the selective alteration of certain hydroxyl groups is important because the resulting acylation products might be useful precursors for the synthesis of new, bioactive products. Moreover, the designed acyl derivatives might exhibit a high antiviral efficacy as versatile intermediates for synthesizing various other antiviral drugs of fundamental relevance.

**Table 1:** Predicted antiviral activities (% inhibition) of cytidine derivatives 2–15, remdesivir, and AZT.

Compounds	General	HBV	HCV	HHV	HIV
**1**	-	-	-	-	-
**2**	56.694	24.616	52.294	39.139	60.776
**3**	50.334	25.168	44.745	68.231	69.760
**4**	51.770	25.164	44.746	65.844	70.174
**5**	7.689	20.217	18.367	48.010	71.148
**6**	5.209	22.034	17.707	32.962	62.485
**7**	8.964	19.478	38.064	46.137	65.673
**8**	54.817	25.306	58.732	51.525	55.146
**9**	2.101	19.505	44.539	44.016	68.671
**10**	2.327	19.694	37.089	45.767	68.719
**11**	50.134	24.790	47.106	60.254	62.961
**12**	4.310	20.681	37.091	43.999	69.047
**13**	3.345	19.376	56.274	81.466	65.758
**14**	47.021	26.981	46.291	75.321	63.214
**15**	61.039	18.367	53.816	61.409	63.427
**Remdesivir**	48.642	22.443	66.968	36.291	69.503
**AZT**	87.038	19.619	24.962	28.728	92.855

HBV = Hepatitis B virus; HCV = Hepatitis C virus; HHV = Human herpesvirus; HIV = Human immunodeficiency virus.

### Structural identification of the designed cytidine derivatives

**Fig 2:**
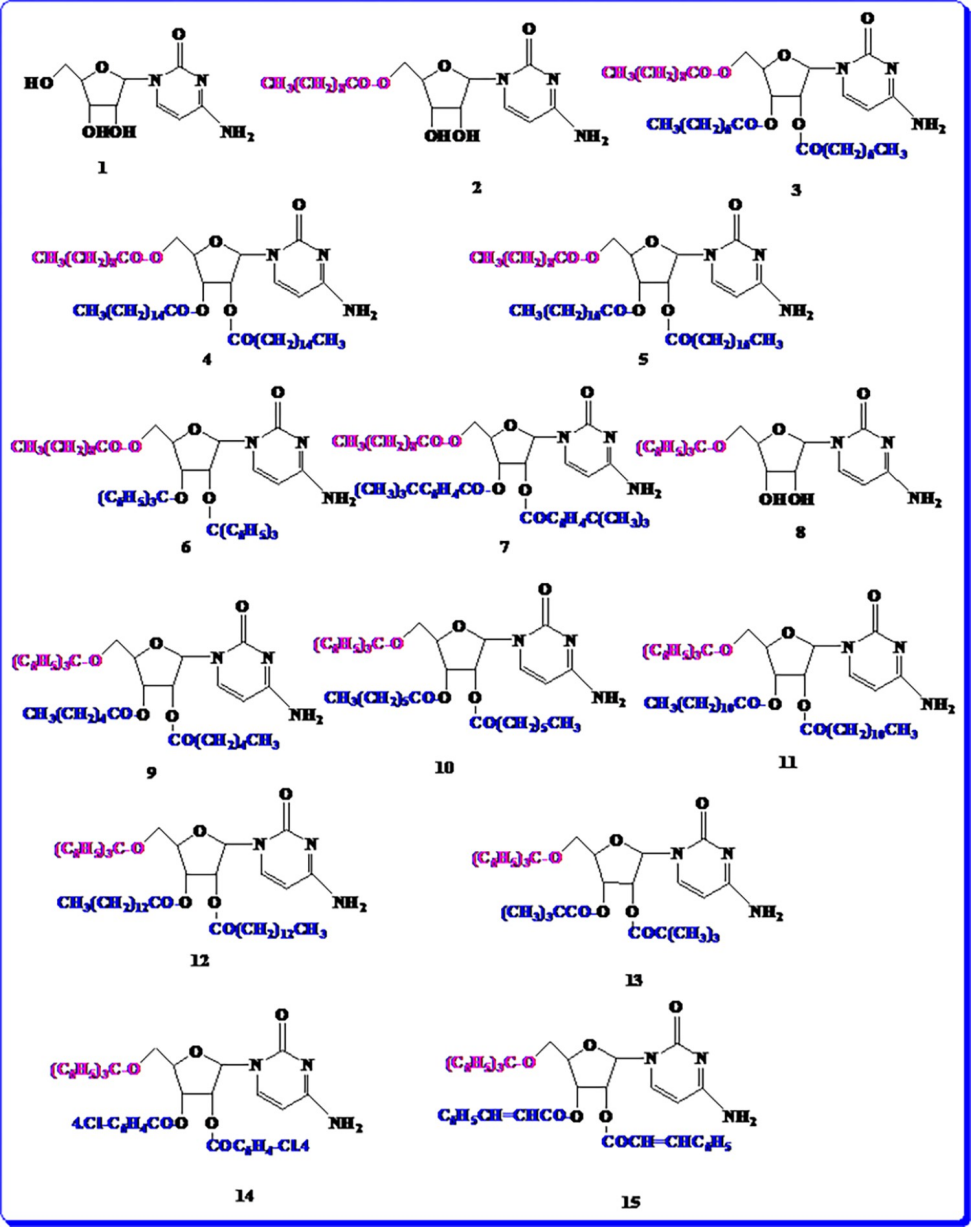
Structures. The designed cytidine (**1**) and its derivatives (**2–15**).

Table S1 in S1 File and Fig 2 and Fig S1 in S1 File present the atomic identification and structural variations of the substituted cytidine derivatives. Different aliphatic (pivaloyl, hexanoyl, octanoyl, decanoyl, lauroyl palmitoyl, myristoyl, and stearoyl and aromatic (4-chlorobenzoyl, cinnamoyl, 4-tert-butylbenzoyl, and trityl) groups were subjected to the hydroxyl (–OH) group modification of cytidine for investigating the variations in biological activities.

### Antiviral activity prediction

When considerable antimicrobial and anti-carcinogenic activities were acquired, we anticipated the antiviral activities of cytidine derivatives (**2–15)** and compared them with those of azidothymidine (AZT, antiviral drug) and remdesivir (COVID-19 drug) by using an antiviral application (Table 1) [57].

The predicted antiviral activities revealed that the modified cytidine derivatives (**2–15**) exhibit potential antiviral efficacy compared with their parent molecules. The aliphatic derivatives (**2–4**) and aromatic derivatives (**8**, **11**, **14**, and **15**) exhibited more promising scores than aliphatic derivatives (**3–5**) along with standard drugs remdesivir and azidothymidine (AZT).

### Molecular electrostatic potential (MESP)

The molecular electrostatic potential (MESP) map indicates the total charge of electrons and nuclei and gives some idea about the nature of electronegativity, partial charge, dipole moment, and chemical reactivity of the molecule. In the computer-aided drug design, atomic charges are employed to investigate the connectivity between the structure and biological activity of drugs. MESP is globally used as a reactivity map displaying the most suitable regions for the electrophilic and nucleophilic attacks of charged-point-like reagents on organic molecules [58].

**Fig 3:**
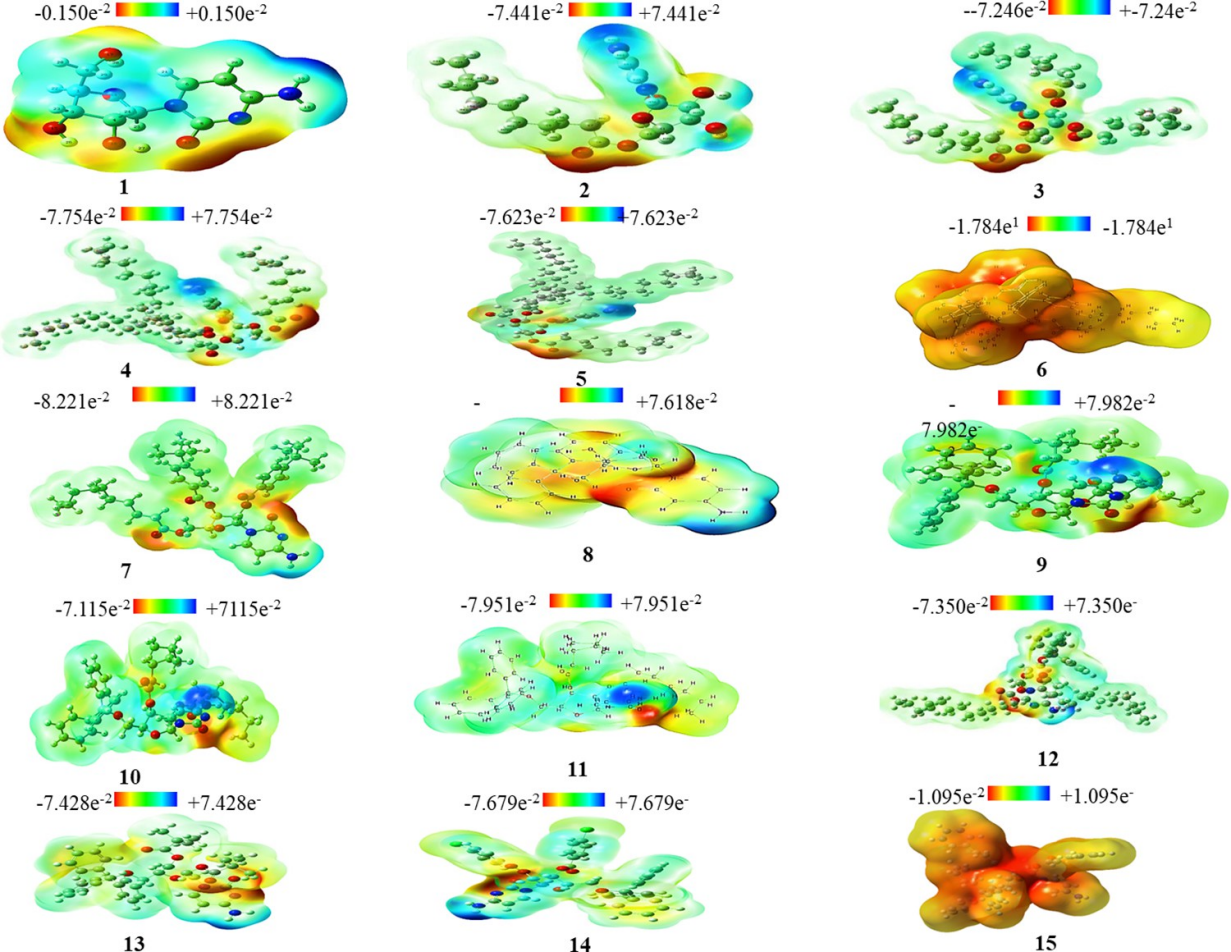
MESP map. Cytidine (**1**) and its derivatives (**2–15**).

MESP helps interpret the biological recognition process and hydrogen bonding interactions [59]. The counter map of MESP provides a simple approach to predicting how different geometries can interact. The MESP of the title compound was obtained based on B3LYP with the basis set B3LYP/6-31G++ optimized results (Fig 3). MESP is important because it simultaneously displays the molecular size and shape and positive, negative, and neutral electrostatic potential regions for color grading and is useful for studying molecular structures with the physicochemical property relationship [60]. From MEP map maximum negative potentiality has been found in the oxygen atom and the highest positive potentiality has been found for the hydrogen atom. MESP was calculated to determine the reactive sites for the electrophilic and nucleophilic attacks of the optimized structure of cytidine derivatives (**2–15**). The red, blue, and green colors represent the maximum negative area favorable for electrophilic attacks, maximum positive area favorable for nucleophilic attacks, and zero potential areas, respectively.

### Molecular docking simulation

**Fig 4:**
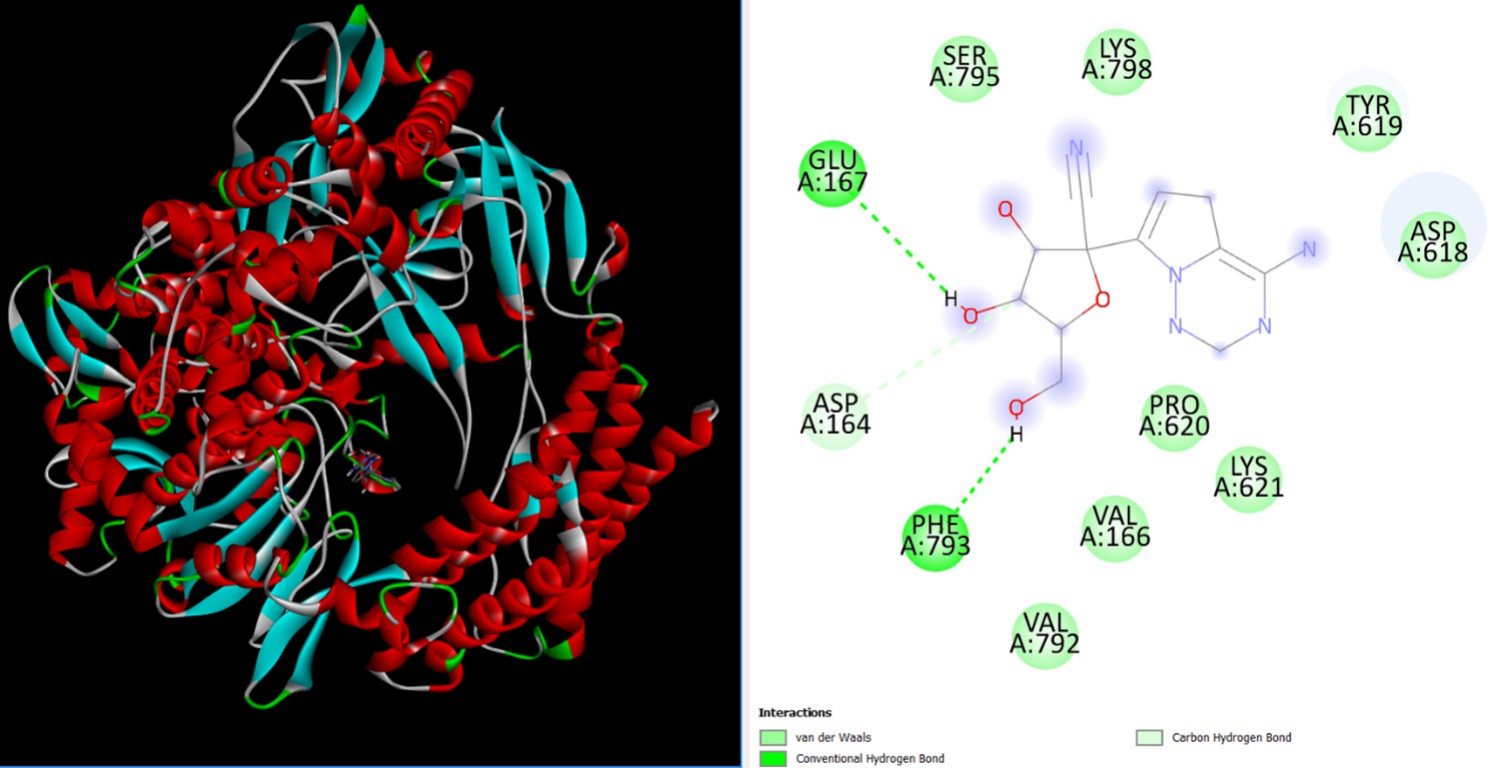
Binding conformation. Interactions of control molecule with the enzyme.

**Fig 5:**
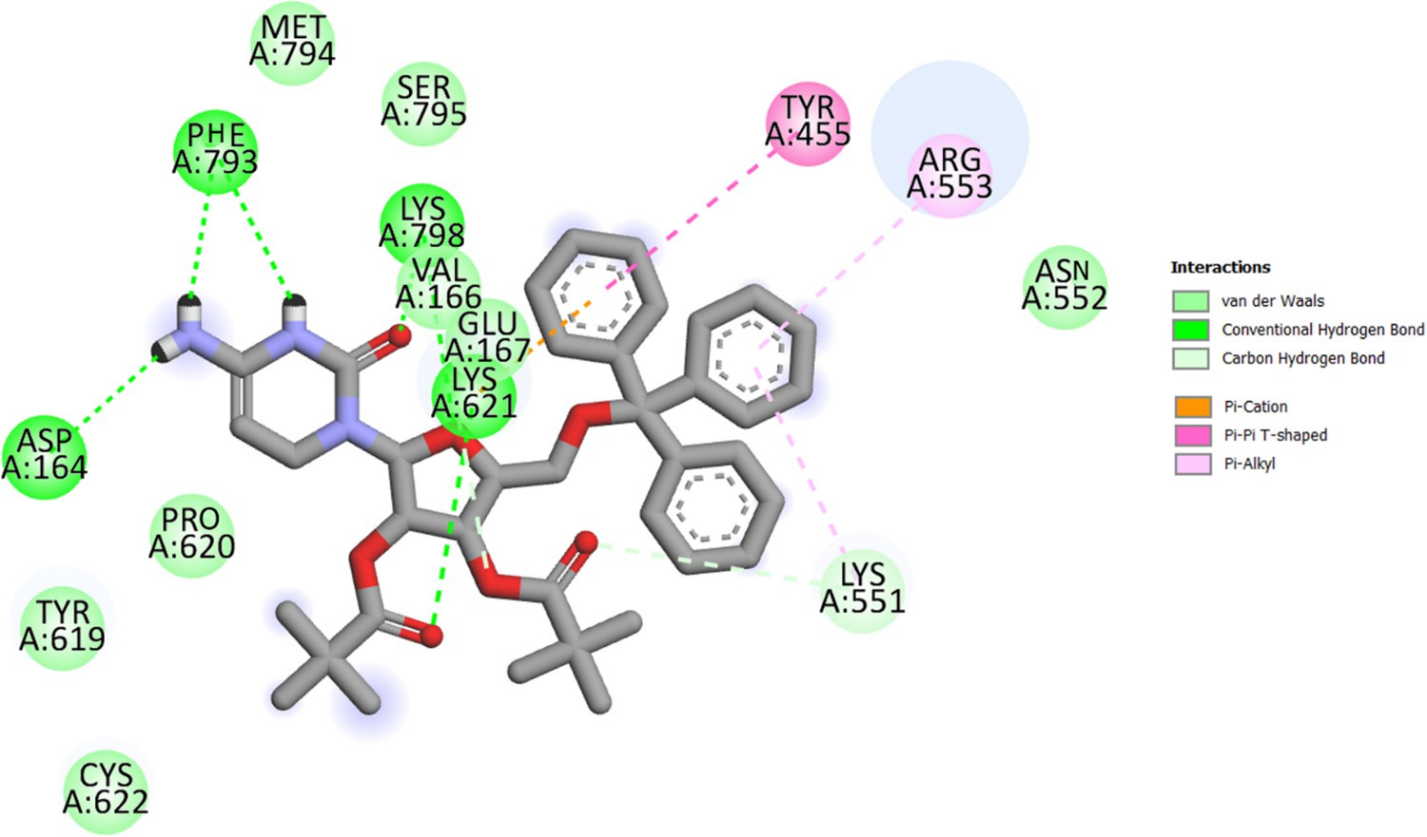
Chemical interactions. Derivatives (**13**) with the active site of SARS-CoV-2 (PDB: 6M71) were performed by Discovery Studio.

**Fig 6:**
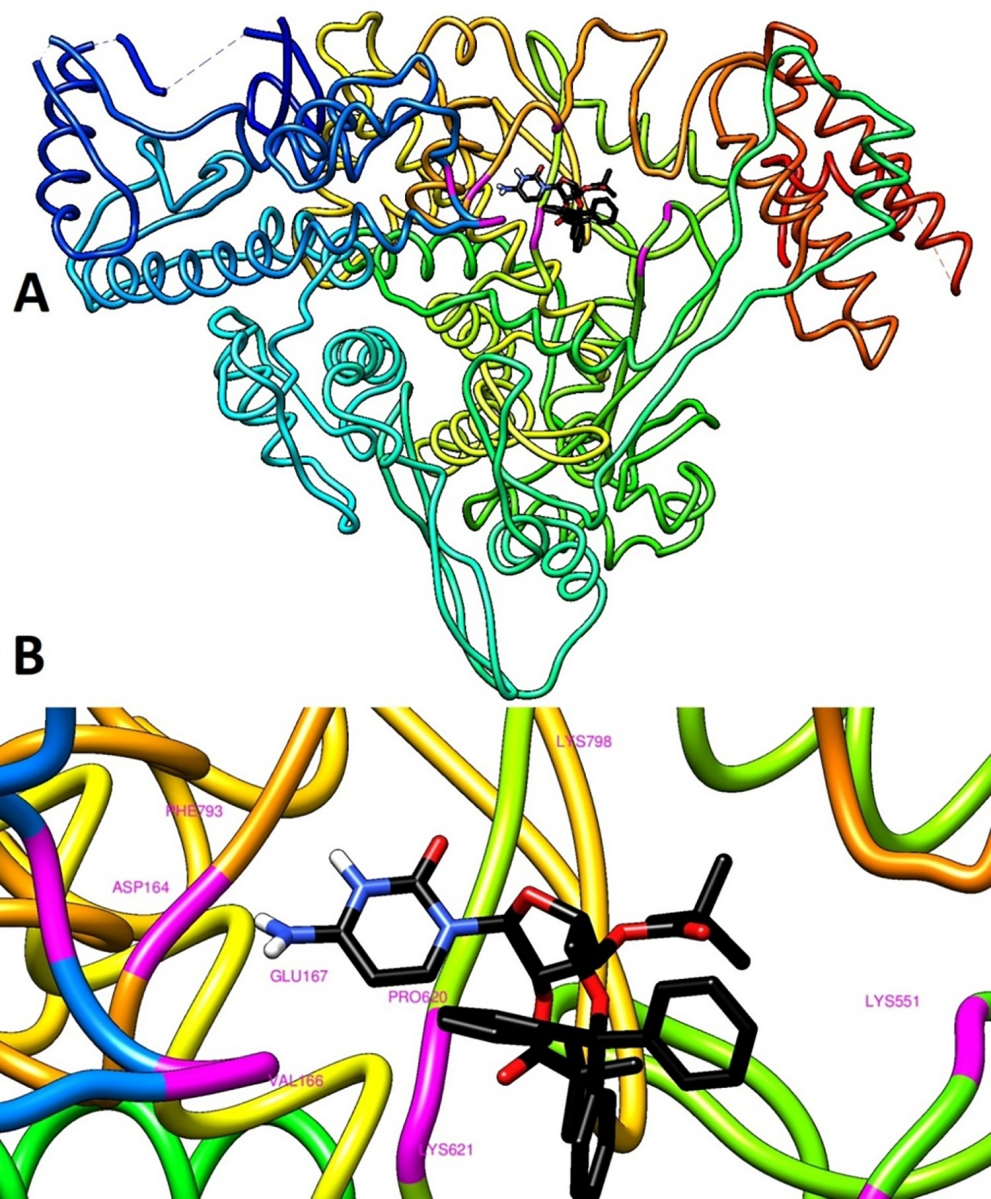
(**A**) Docked 3D pose. Derivative **13** with RdRp (PDB: 6M71). (**B**) The close viewer of key active site residues involved in interactions with derivative **13**.

In structural biology and computer-aided drug design, molecular docking is an important technique. The key aim of molecular docking is to determine the potential binding geometries of a putative ligand of a known 3D structure with a target protein. To validate docking studies, co-crystallized remdesivir was redocked with RdRp enzymes. The binding mode and interactions of the control remdesivir with the enzymes are given in Fig 4. In this study, several cytidine derivatives were studied *in silico* to determine their possible binding energies and interaction modes at the active site of SARS-CoV-2 RdRp (Table 2). Table 2 presents the estimated average binding energies of compounds with the enzymes (Fig S2 in S1 File). According to the docking screening results, eight derivatives (**6**–**10** and **13**–**15**) with the strongest binding energies were selected to describe the binding mode of cytidine inhibitors. Comparatively, the aromatic derivatives exhibited better binding scores than the aliphatic derivatives. Fig 5 illustrates 2D interactions between the strongest inhibitor (compound **13**) and active site residues of RdRp. The interactions included hydrophobic contacts, Van der Waals interactions, hydrogen bonding, electrostatic interactions, carbonyl interactions, and a specific atom-aromatic ring. Fig 6 present the docked conformation of the most active molecule (**13**) based on the docking studies. The results showed derivative (**13**) as the most promising ligand (−11.2 kcal/mol) that bound with SARS-CoV-2 RdRp through hydrophobic bonding and many hydrogen interactions. The binding site is located in the hydrophobic cleft bordered by amino acid residues VAL166, GLU167, SER795, PRO620, TYR619, CYS622, and MET794. Four hydrogen bond contacts occur with four different amino acids, ASP164, LYS621, LYS798, and PHE793 at distances of 2.526, 2.814, 2.417, and 2.282 Å, respectively. In a previous study, it has been demonstrated that Ornipression showed binding with the same active pocket reported herein and interacting with the same set of residues at a close distance. The research also unveiled Nacartocin as a potent binder of the active pocket predicted in this study [61]. In another work, Cefuroxime, Tenofovir, Ribavirin, Sofosbuvir, Galidesivir, Remdesivir, and Hydroxychloroquine were reported to docked and interact with the same set of residues of RdRp shown in this study [62]. Compounds (**13** and **14**) have an additional benzene ring in cytidine, providing a high density of electrons in the molecule and the highest binding score [63]. These results indicated that modification of the–OH group along with long carbon chains/aromatic ring molecules led to an increase in the binding affinity. The addition of hetero groups such as Br caused some fluctuations in binding affinities; however, modification with halogenated aromatic rings led to an increase in the binding affinity. The docked pose showed that the drug molecules bind within the active site of the SARS-CoV-2 RdRp.

**Table 2:** The binding energy of the cytidine derivatives against RdRp.

RdRp (pdb: 6M71)					RdRp (pdb: 6M71)				
Compounds	Binding affinity	No. of hydrogen bond	No. of hydrophobic bond	NBI	Compounds	Binding affinity	No. of hydrogen bond	No. of hydrophobic bond	NBI
**1**	-6.1	5	2	H, A	**2**	-4.0	3	1	H, PDH, PA
**3**	-5.8	3	3	H, PAn, A, PA	**4**	-5.1	2	4	H, A
**5**	-7.5	5	12	C, A, PA	**6**	-6.1	3	4	PPT, PA
**7**	-8.6	3	8	H, PS, A	**8**	-8.4	3	3	H, PAn, PA
**9**	-8.4	4	9	H, PS, APS, A, PA	**10**	-7.0	5	8	H, PS, APS, A, PA
**11**	-5.8	3	7	H, PS, PPS, PPT, PA	**12**	-5.2	1	6	H, PS, APS, A, PA
**13**	-11.0	4	9	H, PS, APS, PA	**14**	-10.2	6	8	H, PS, APS, A, PA
**15**	-7.0	2	6	H, PS, PA	Remdesivir	10.0	4	10	H, PS, APS, A, PA

NBI: Nonbonding interaction; H = Conventional hydrogen bond; C = Carbon–hydrogen bond; A = Alkyl; PA = π-alkyl; PAn = Pi-anion; APS = Amide pi-stacked; PDH = π-donor hydrogen bond; PPS = π–π stacked; and PPT = π–π T-shaped.

Parent molecule cytidine (**1**) interacted with the key residues of the main protease; LYS621 and PRO620 through hydrogen bonding within a close bond distance (2.17 Å). Additionally, LYS798, PHY793, and LYS551 interactions were observed, and interaction with LYS621 showed a shorter bond distance (2.277Å) due to the unique interaction of the branched alkyl chain with the cytosine base.

These derivatives exhibited diverse nonbinding interactions, such as π–anion, π–donor hydrogen bonds, amide pi-stacked, π–π stacked, and π–π T-shaped interactions, with the active sites of the main protease.

The aromatic substituents led to an increase in the binding energies of derivatives; **7** to **10** = −7.4, −7.4, −7.4, and −7.0 kcal/mol, respectively, and **13–15 =** −8, −6.2, and −9.2 kcal/mol, respectively. These derivatives interacted with the similar residues of the RdRp: PRO620, LYS798, PHY793, LYS55, and LYS621. Amongst all the residues, LYS621 exhibited a minimum bond distance of < 2.068 Å. These results revealed that thanks to the high electron density, aromatic substituents can easily lead to an increase in the binding and antiviral abilities of the cytidine derivatives. Along with PRO620, all the derivatives showed maximum π–π interactions with ASN552. PRO620 is considered the principal component of PPS, APS, and PPT, which are responsible for the accessibility of small molecules to the enzyme’s active site. Binding energies and binding modes were observed for derivatives (**7–10** and **13–15**) due to significant hydrogen bonding. The alterations of the–OH group in cytidine exalted the π–π interactions with the amino acid chain at the binding site, and their polarity improvement resulted in hydrogen bond formation.

Ten commercial medicines possibly form H-bonds with the key residues of the 2019-nCoV RdRp [64]. H-bonds play a vital role in shaping the specificity of ligand binding with receptors, drug design in chemical and biological processes, molecular recognition, and biological activity. The blind docking study of all the cytidine derivatives with the SARS-CoV-2 RdRp revealed that the molecules were generally surrounded by the aforementioned residues, which is similar to the arrangement in standard drugs. This finding suggested that this molecule may prevent the viral replication of SARS-CoV-2.

### Molecular dynamics simulations

**Fig 7:**
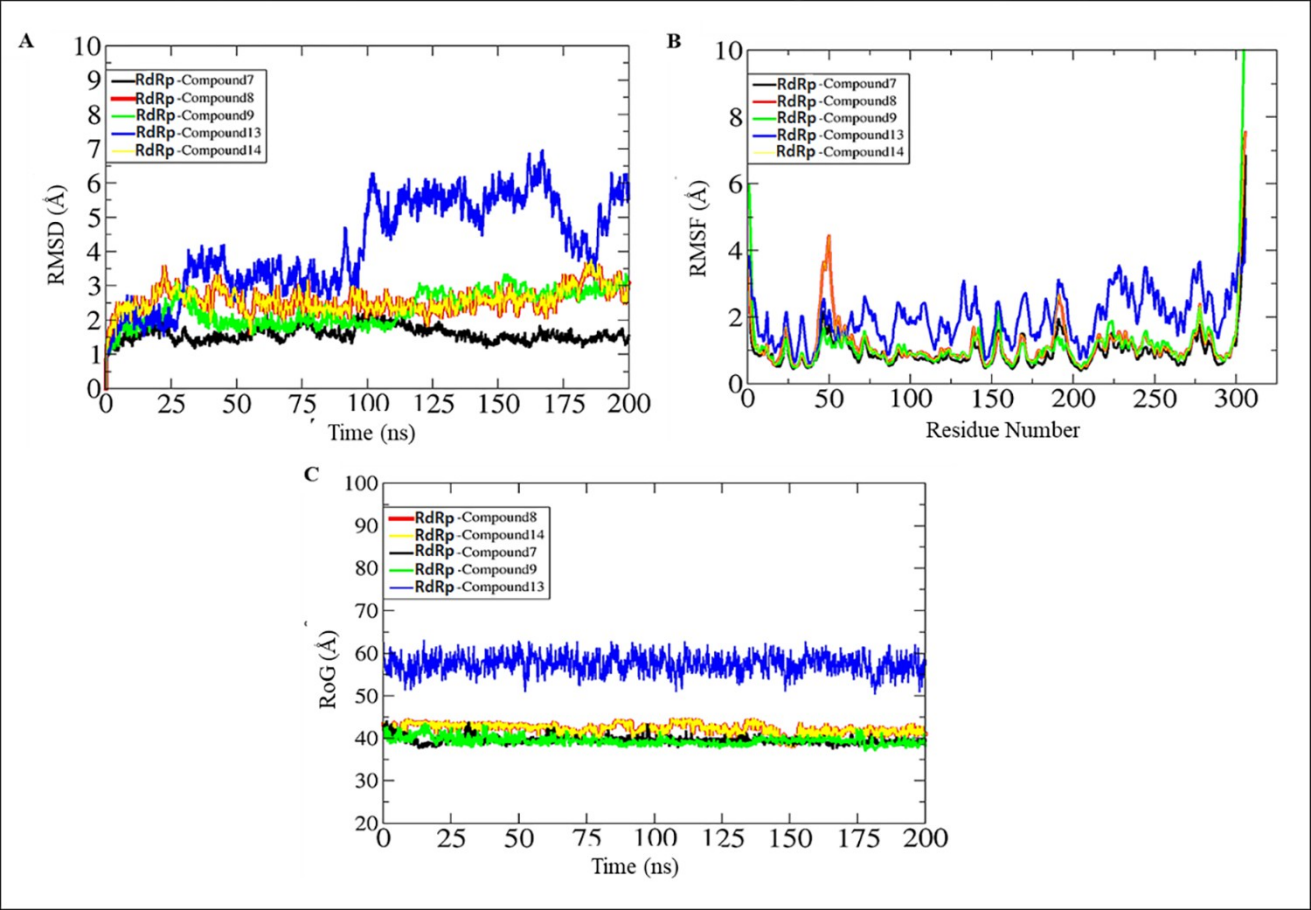
Backbone structural deviations. Analysis based on molecular dynamics simulations. A. RMSD, B. RMSF and C. RoG.

**Fig 8:**
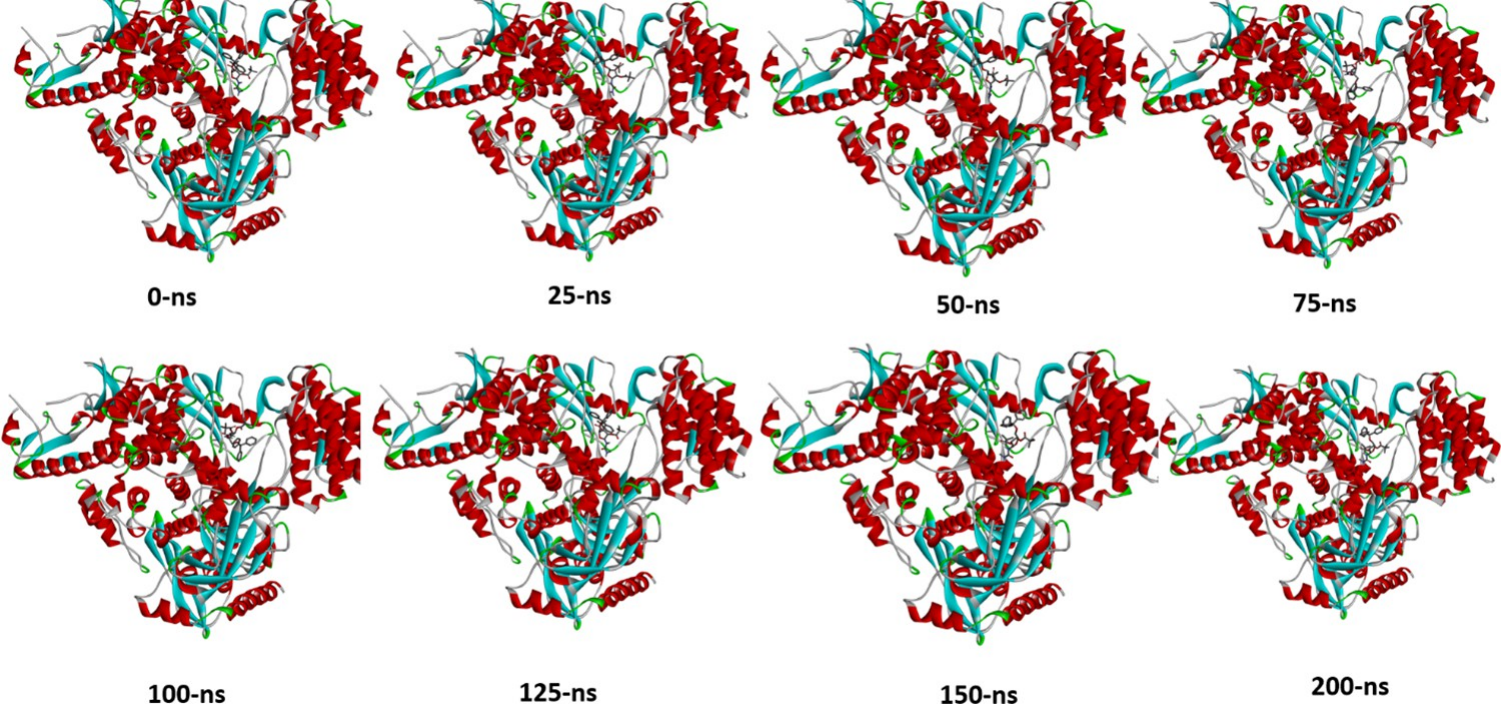
Molecular dynamic simulation. Snapshots of compound 13 with the enzyme at different nanoseconds.

The conformational dynamics and stability of RdRp-compound complexes were evaluated using root mean square deviations (RMSD) based on 200 ns of molecular dynamics simulation trajectories [65]. The carbon alpha RMSD for all studied systems is illustrated in Fig 7A. As can be seen that most of the systems are in equilibrium with no major alterations in the plots. The RdRp -compound 7, RdRp -compound 8, RdRp -compound 9, and RdRp -compound 14 systems were reported in stable dynamics and the RMSD was within a stable range. Initial, minor deviations were spotted that upon an investigation of trajectories disclosed ligands induced pressure on the receptor loops. Once, the binding conformation achieved stability no further conformation changes in the receptors were noticed. The RdRp-compound 13 system was stable in the initial 25 ns, followed by constant conformation deviations till 100 ns. From onward to 175 ns, the system remained relatively stable. Toward the end time, the system reported reduced RMSD and achieved a minor constant RMSD. This behavior of the system can be explained by the regularly changed binding pose of the compound at the binding pocket of the enzyme, however, the binding affinity is stronger and still produced good intermolecular interactions compared to the rest of the compounds. The different snapshot of compound 13 with the enzyme is given in Fig 8. Thus, a lengthy production run is needed to decipher the dynamic stability of this complex. The receptor residues can be seen in a good RMSF (Fig 7B) range except for the C and N terminal residues which by nature are more flexible due to the absence of any fixed 3D secondary structures. The majority of the enzyme residues are stable and the active pocket is in good stability. Lastly, the radius of gyration (RoG) was investigated to shed light on the complex compactness and validation of the systems RMSD. Complementing RMSD analysis, RoG predicted all systems in very good equilibrium with no profound deviations unraveled (Fig 7C). The mean RoG of all systems is around the 50–80 Å range.

### MMGBSA binding free energies

The MMGBSA binding free energy estimation is highly useful in drug design as the method is modest in energy use and more reliable than the docking method [66]. In total, 1000 frames were considered from simulation trajectories for estimating different energy terms. All the systems were revealed to have highly favorable net binding free energies. From energy term-wise, the Van der Waals energy of the systems was found highly dominating followed by electrostatic energy. This means that in RdRp-drug interactions, both hydrophobic and hydrophilic interactions were key in stable docked conformation and blocking the enzyme active pocket. The polar solvation energy was found less critical in overall complex stabilization. While the non-polar solvation energy seems favorable during the compound’s interaction with the enzyme. The net binding energy of the systems is as follows; RdRp-Compound **7** (-83.4 kcal/mol), RdRp-Compound **8** (-94.71 kcal/mol), RdRp-Compound **9** (-78.00 kcal/mol), RdRp-Compound **13** (-78.98 kcal/mol) and RdRp-Compound **14** (-82.93 kcal/mol). The details of the contribution of each energy term in complex formation are tabulated in Table S2 in S1 File.

### Biological evaluation

The inhibition capacity of cytidine-like nucleosides against SARS-CoV-2 RdRp has been explored *in vitro* [31, 32]. Because SARS-CoV and SARS-CoV-2 viruses are highly similar, we investigated the in-silico behavior of the cytidine derivatives toward SARS-CoV-2 RdRp. Selecting this protein as the target led to considerable advances in antiviral treatment because it participates in the proteolytic processing of polyproteins replication. Consequently, it plays a key role in the expression and replication of viral genes. Therefore, the inhibition of this enzyme hampered the replication of the viral genome and multiplication of SARS-CoV-2. Nucleoside derivatives that can inhibit SARS-CoV RdRp may inhibit SARS-CoV-2 RdRp in the same manner due to their high-sequence identity.

### Pharmacokinetic profile and molecular radar

To predict the pharmacokinetic properties (Table 3), such as solubility, lipophilicity, and toxicity of the compounds, we used the pkCSM ADMET descriptor algorithm protocol. Drug absorption depends on various factors, including membrane permeability indicated by the cell line of colon cancer (Caco-2), intestinal absorption, skin permeability thresholds, substrate, and P-glycoprotein inhibitors.

**Table 3:** Prediction in silico of absorption of cytidine derivatives.

Compounds	Water solubility	Lipophilicity	Caco2 permeability	Skin permeability
	(log mol/L)	(Consensus Log *P*_o/w_)		
**1**	-1.689	-1.77	0.025	-2.745
**2**	-3.166	1.35	0.233	-2.749
**3**	-4.042	6.47	0.263	-2.735
**4**	-2.977	12.28	-0.807	-2.735
**5**	-2.920	13.47	-0.917	-2.735
**6**	-2.892	9.15	-1.732	-2.735
**7**	-3.616	6.95	-0.355	-2.735
**8**	-3.728	2.44	0.559	-2.735
**9**	-4.363	5.83	-0.590	-2.735
**10**	-4.077	6.65	-0.598	-2.735
**11**	-3.075	9.86	-0.625	-2.735
**12**	-2.951	11.23	-0.627	-2.735
**13**	-4.458	5.05	-0.118	-2.735
**14**	-2.931	6.44	0.769	-2.735
**15**	-2.950	5.94	0.870	-2.735

**Fig 9:**
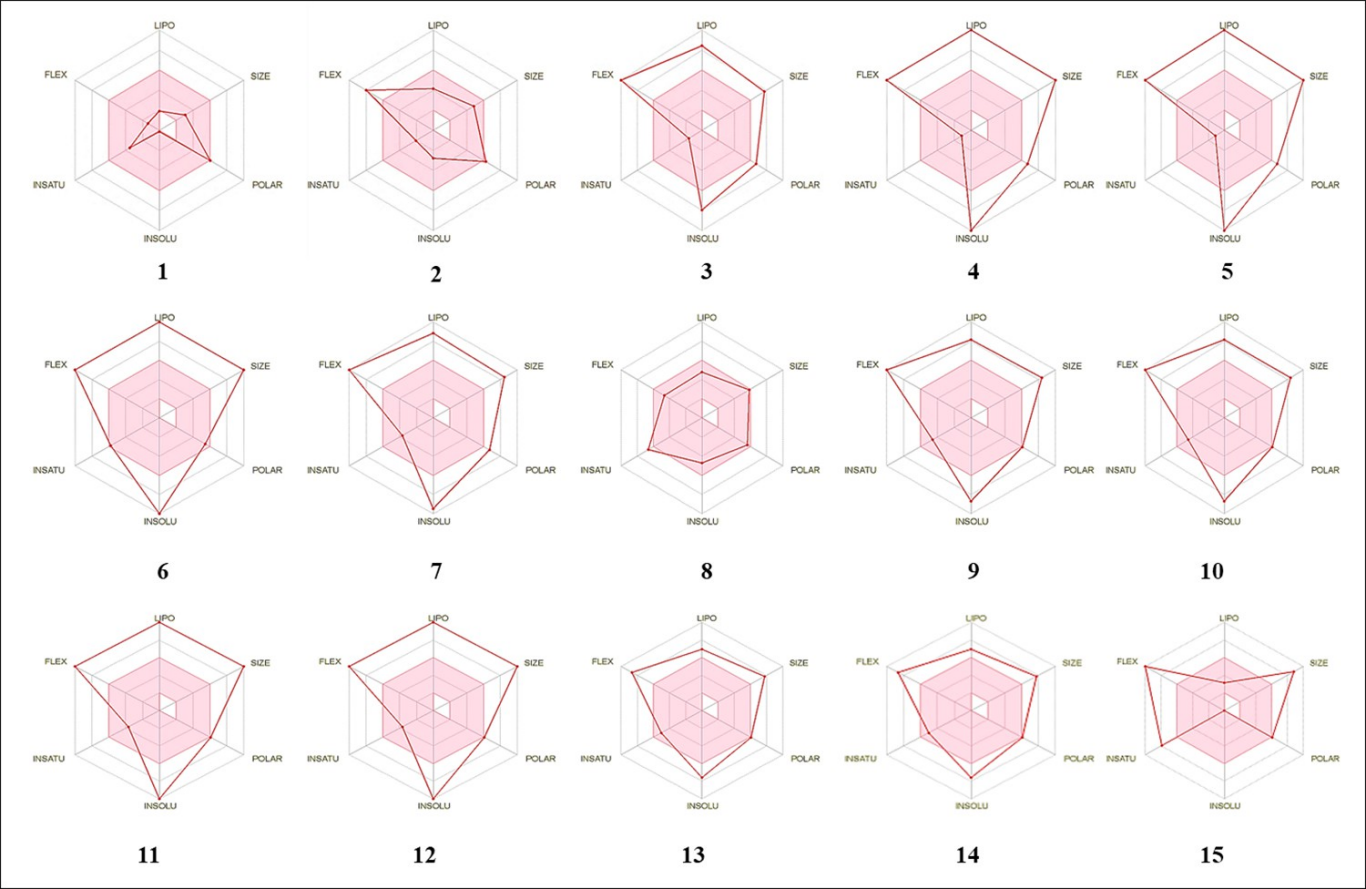
Bioactivity radar. Charts of the cytidine derivatives where FLEX: Flexibility, LIPO: Lipophilicity, INSATU: Unsaturation. and INSOLU: Insolubility.

All the derivatives showed excellent lipophilicity with values of −1.35 to 13.47 (Table 3). Skin permeability is an important factor for drug efficacy improvement, especially in the development of transdermal drug delivery. A molecule barely penetrates the skin if log Kp is more than −2.5 cm/h [67]. The skin permeability Kp of the cytidine derivatives is −2.731 cm/h (<−2.5) (Table 3). Therefore, all the presented derivatives exhibit high skin penetrability. In the pkCSM predictive model, high Caco-2 permeability is translated into the predicted log Papp values > 0.90 cm/s. The value of Caco-2 permeability (log Papp) of the cytidine derivatives ranges from −4.3 to −2.4 cm/s, log Papp < 0.9 cm/s (Table 3); thus, these derivatives exhibit a low Caco-2 permeability. Molecular radar is a crucial QSAR factor exhibiting the molecular volume of compounds. Fig 9 illustrates the physicochemical radar of all the cytidine derivatives and reveals the promising QSAR features of the designed compounds.

To discover oral administrative drugs, solubility is a major descriptor. High water solubility is useful to deliver active ingredients in a sufficient quantity with small volumes of pharmaceutical dosage. These water solubility values are presented as log (mol/L) (insoluble ≤ −10 < poorly soluble < −6 < moderately soluble < −4 < soluble < −2 < very soluble < 0 ≤ highly soluble). The tested compounds are soluble (Table 3). The bioactivity score of lead analogs is predicted with a combination of GPCR, ion channel modulator, kinase inhibitors, nuclear receptor ligands, protease inhibitor, and enzyme inhibitors, which has been employed to identify the efficacy of molecules to qualify for drug development. The larger the bioactivity score the higher probability of the specific molecule being active. If the bioactivity score of molecules is greater than 0.00, has promising biological activities and a score ranging (0.50–0.00) are taken to be moderately active and if the value is less than −0.50 it is presumed to be inactive. The bioactivity score of all the designed cytidine derivatives is displayed in Table 4. The bioactivity score values obtained showed that derivatives (**2**, **3,** and **8**) followed the promising efficacy.

**Table 4:** Determination of drug-likeness score of cytidine derivatives through Molinspiration cheminformatics online server.

Compounds	GPCR ligand	Ion channel modulator	Kinase inhibitor	Nuclear receptor ligand	Protease inhibitor	Enzyme inhibitor
**1**	0.73	0.12	0.37	-1.65	-0.07	1.12
**2**	0.60	0.04	0.23	-0.82	0.21	0.74
**3**	-0.01	0.81	-0.39	-1.03	-0.06	-0.10
**4**	-2.62	-3.51	-3.25	-3.61	-2.12	-2.87
**5**	-3.21	-3.65	-3.58	-3.73	-2.92	-3.35
**6**	-3.23	-3.67	-3.59	-3.72	-2.91	-3.29
**7**	-0.71	-1.79	-1.31	-1.86	-0.57	-0.96
**8**	0.38	-0.14	0.19	-0.52	0.11	0.70
**9**	-0.45	-1.53	-0.98	-1.43	-0.33	-0.49
**10**	-0.72	-1.90	-1.32	-1.78	-0.53	-0.81
**11**	-2.64	-3.54	-3.29	-3.57	-2.12	-2.76
**12**	-3.24	-3.67	-3.59	-3.71	-3.92	-3.25
**13**	-0.25	-1.15	-0.65	-1.10	-0.16	-0.21
**14**	-1.10	-2.34	-1.71	-2.24	-0.87	-1.26
**15**	-1.42	-2.79	-2.15	-2.60	-1.09	-1.60

### Calculation of QSAR and pIC_50_

The quantitative structure-activity relationship (QSAR) is a computational modeling approach for revealing correlations among the structural characteristics of chemical substances and biological activity. To complete the calculated QSAR and pIC50 value, we took help from a free web tool called Chemdesk and takes the required value including Chiv5, MRVSA9, PEOEVSA5, GATSv4, etc. After that, the multiple linear regression (MLR) equations are utilized to obtain the QSAR and pIC50 values [68]. Our finding compound has been shown and meets all the criteria and different QSAR and pIC50. The range of the QSAR and pIC50 lowest value was obtained at 3.22 whereas the highest value has obtained at 6.28 (Table 5).

**Table 5:** Data of QSAR.

Entry	Chiv5	(bcutm1)	(MRVSA9)	(MRVSA6)	(PEOEVSA5)	GATSv4	PIC50
**1**	0.534	2.074	0.000	0.00	0.00	0.91	3.78
**2**	1.002	3.284	4.274	0.00	63.232	0.97	3.22
**3**	2.510	4.521	18.101	84.35	75.007	1.01	3.88
**4**	2.857	4.803	27.252	92.40	83.851	1.13	4.64
**5**	4.642	4.251	29.319	101.27	83.851	1.37	5.08
**6**	6.204	3.658	45.297	100.09	103.659	1.42	5.32
**7**	5.541	5.270	38.231	105.53	159.668	1.55	6.00
**8**	0.357	1.287	5.312	0.00	61.522	0.87	3.56
**9**	3.714	3.280	23.051	90.91	120.474	1.00	4.99
**10**	5.916	4.281	31.594	100.73	131.976	1.19	5.37
**11**	5.284	4.005	36.024	107.87	139.817	1.33	5.86
**12**	7.251	5.299	42.981	103.61	139.817	1.37	6.28
**13**	6.200	3.274	49.993	97.39	169.210	1.46	4.11
**14**	6.842	4.257	25.147	109.11	124.568	1.53	4.57
**15**	5.107	3.022	32.247	92.24	111.027	1.41	5.78

### POM analyses

**Fig 10:**
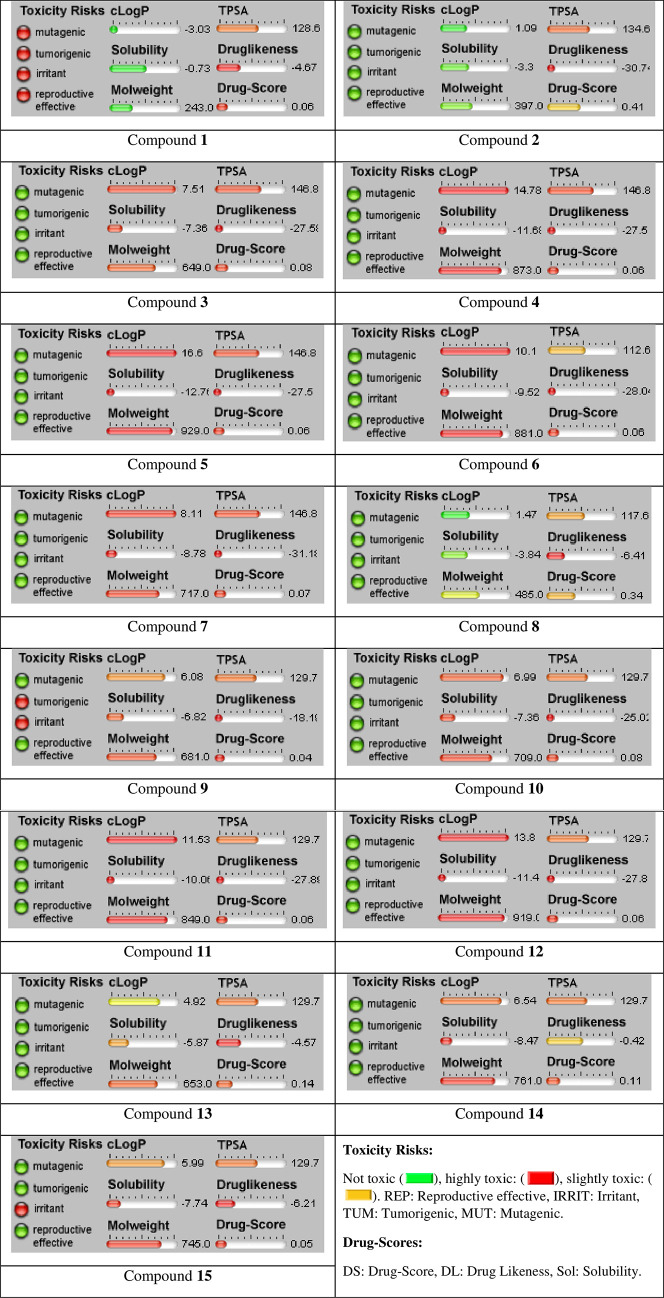
Determination of drug-likeness. Score of cytidine derivatives (**1–15**) through Osiris cheminformatics online server.

**Fig 11:**
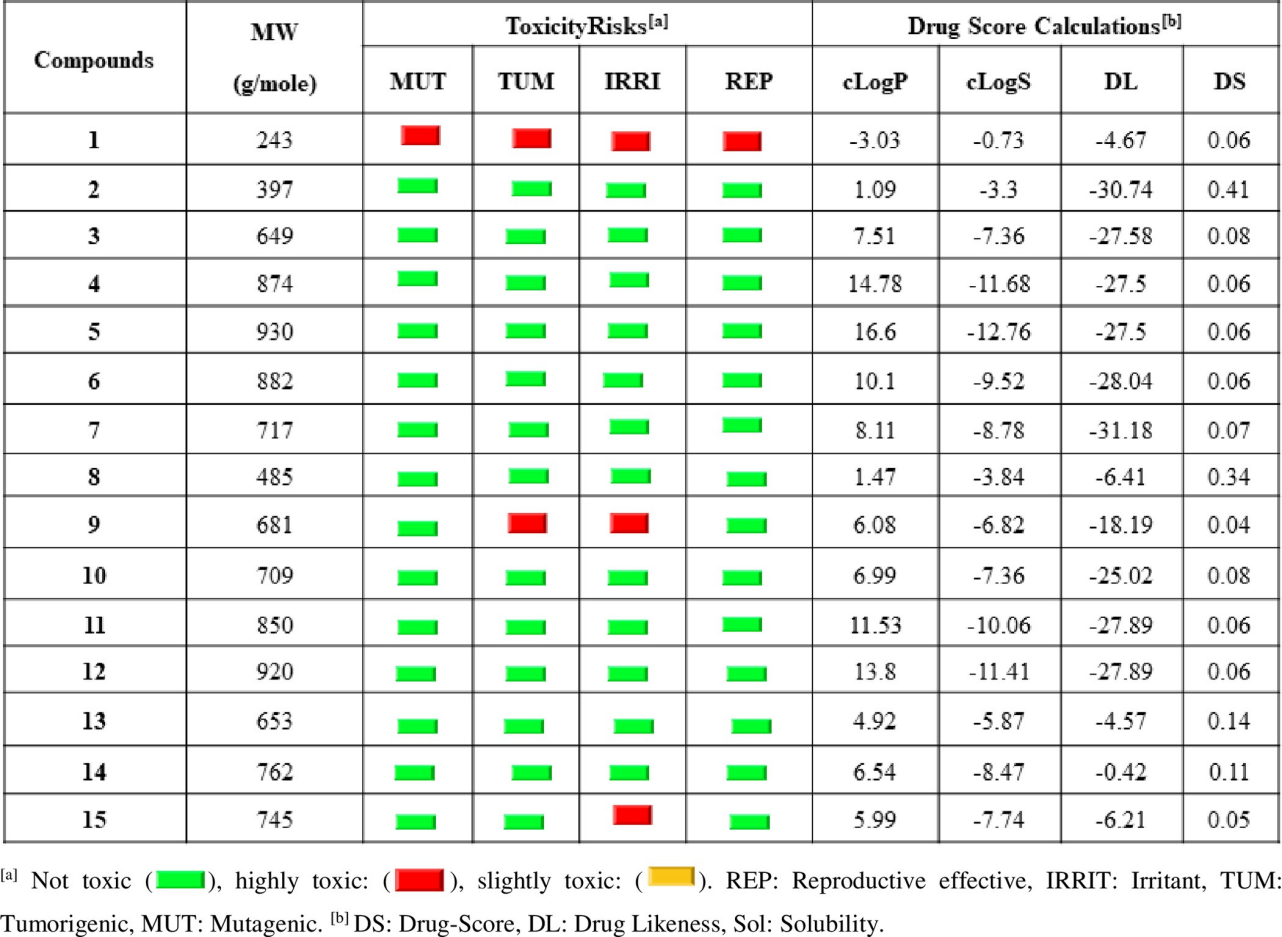
Comparison. Toxicity risks and drug score calculations of compounds **1–15**.

As Molinspiration analysis is given above (Table 4), we proceed with Osiris calculations. Figs 10 and 11 show the toxicity risks and drug score calculations of compounds **1–15**. It seems that only 3/15 of compounds present side effects. This led us to get more good scores.

**Fig 12:**
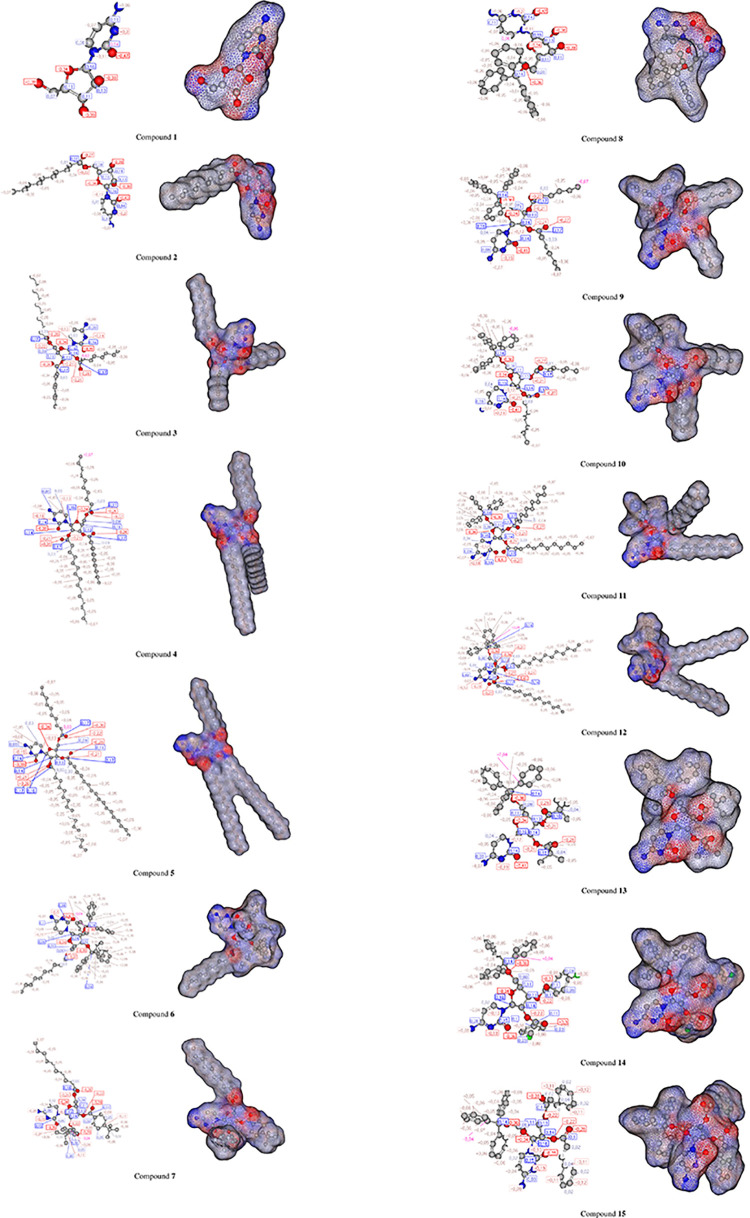
Atomic charge. Calculations of compounds **1–15**.

**Fig 13:**
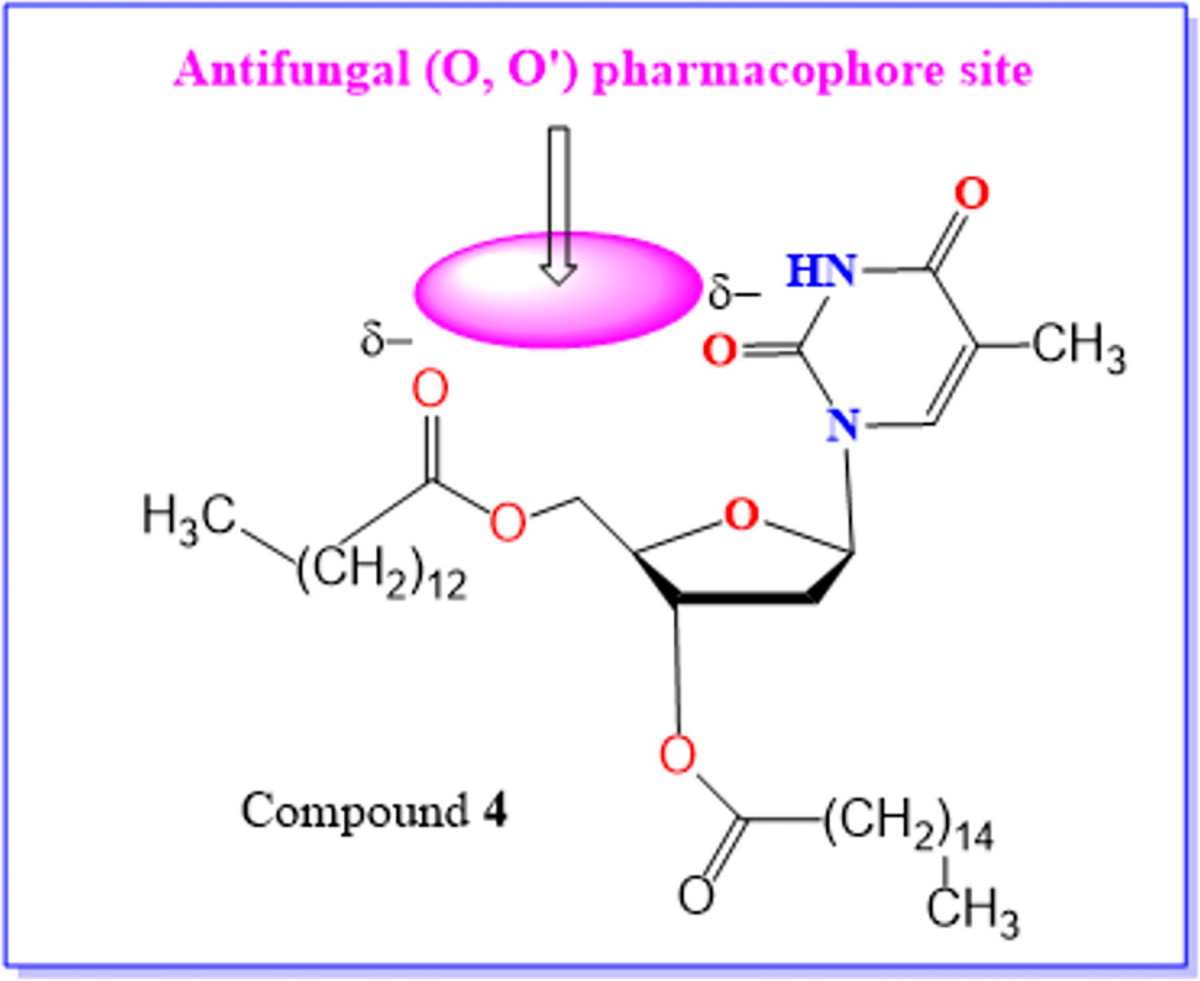
Identification. Antifungal/antiviral pharmacophore site of compound **4** [69–75].

The atomic charge calculations (Fig 12) indicate clearly that most compounds **1–15** possess an (O^δ−^−−−−O’^δ−^) antifungal/antiviral pharmacophore site. For this reason, most of the hits are more antifungal than antiviral agents so, it has been identified as an antifungal/antiviral pharmacophore site of compound **4** (Fig 13).

## Conclusions

We conducted a computational study to identify the new inhibitors of anti-SARS-CoV-2; molecular docking was studied for a series of nucleoside (cytidine) derivatives, known as anti-SARS-CoV-2 agents. All the cytidine derivatives were successfully analyzed *in silico* for their antiviral activity prediction, MESP calculation, molecular docking, and pharmacokinetics properties. The insertion of various aliphatic and aromatic groups into the cytidine structure can considerably improve their biological and antiviral activity modes. Antiviral prediction indicates that aliphatic (**2–4**) and aromatic (**8**, **11, 14**, and **15**) derivatives exhibit potential antiviral modes. These findings were rationalized through molecular docking, which revealed the excellent antiviral efficacy of the cytidine derivatives. Many derivatives showed outstanding binding energy and binding interactions with SARS-CoV-2 RdRp. Eight cytidine derivatives (**6**–**10** and **13–15**) exhibit *in silico* a potent ability to inhibit SARS-CoV-2. The derivatives **7–9** and **13–14** were further reported to have enhanced dynamic stability as revealed by their uniform RMSD, and RoG profiles. The derivatives were unraveled in stable binding conformation at the docked pocket, engaged by both hydrophobic and hydrophilic interactions, which strengthened interactions as the simulation time proceeded. The snapshots taken from the simulation trajectories revealed the complexes to form strong electrostatic and van der Waals interactions. Pharmacokinetic prediction provided promising results for *in silico* properties, revealing that all the modified compounds exhibit an iRdRpved pharmacokinetic profile. Future *in vitro* and *in vivo* studies should determine whether these derivatives can be drugs candidates used to treat SARS-CoV-2. POM study confirms the predominant antiviral/antifungal profile of most compounds of series **1–15**. This is highly encouraging to screen the antifungal hits as potential antiviral candidates. This investigation revealed that cytidine derivatives having a short aliphatic chain (C5-C10) and aromatic ring exhibited potentiality in all the studied cases. Based on these analyses, derivatives (**7**, **8**, **9**, **13**, and **14**) are suggested as the best choice as an anti-SARS-CoV-2 agent.

## Supporting information

S1 File (DOCX)
