# Supplementary material for: Potential SARS-CoV-2 RdRp inhibitors of cytidine derivatives: Molecular docking, molecular dynamic simulations, ADMET, and POM analyses for the identification of pharmacophore sites
Source: PLoS One. 2022 Nov 28;17(11):e0273256. doi: 10.1371/journal.pone.0273256 (PMC9704642; doi:10.1371/journal.pone.0273256)
Supplement: S1 File — (DOCX) [file pone.0273256.s001.docx]

**Supplementary information**

**Potential SARS-CoV-2 RdRp Inhibitors of Cytidine Derivatives: Molecular Docking,**

**Molecular Dynamic Simulations, ADMET, and POM Analyses for the Identification of Pharmacophore Sites**

Sarkar M. A. Kawsar^*,a,^ Mohammed A. Hosen^a^, Sajjad Ahmad^b^, Youness El Bakri^*,c^, Hamid Laaroussi^d^, Taibi Ben Hadda^d,e^, Faisal A. Almalki^e^ , Yasuhiro Ozeki^f^, Souraya Goumri-Said^*,g,^

*^a^Laboratory of Carbohydrate and Nucleoside Chemistry (LCNC), Department of Chemistry, Faculty of Science, University of Chittagong, Chittagong-4331, Bangladesh.*

*^b^Department of Health and Biological Sciences, Abasyn University, Peshawar 25000, Pakistan.*

*^c^Department of Theoretical and Applied Chemistry, South Ural State University, Lenin prospect 76, Chelyabinsk, 454080, Russian Federation.*

*^d^Laboratory of Applied Chemistry & Environment, Faculty of Sciences, Mohammed Premier University, MB 524, 60000 Oujda, Morocco.*

*^e^Department of Pharmaceutical Chemistry, Faculty of Pharmacy, Umm AlQura University, Makkah 21955, Saudi Arabia.*

*^f^School of Sciences, Yokohama City University, 22-2, Seto, Kanazawa-ku, Yokohama 236-0027, Japan.*

*^g^College of Science, Physics Department, Alfaisal University, P.O. Box 50927, Riyadh, 11533, Saudi Arabia.*

*****Corresponding authors: E-mail addresses:

Prof. Sarkar M. A. Kawsar, [akawsar@cu.ac.bd](about:blank)

Dr. Youness El Bakri, yns.elbakri@gmail.com

Prof. Souraya Goumri-Said, [sosaid@alfaisal.edu](about:blank)


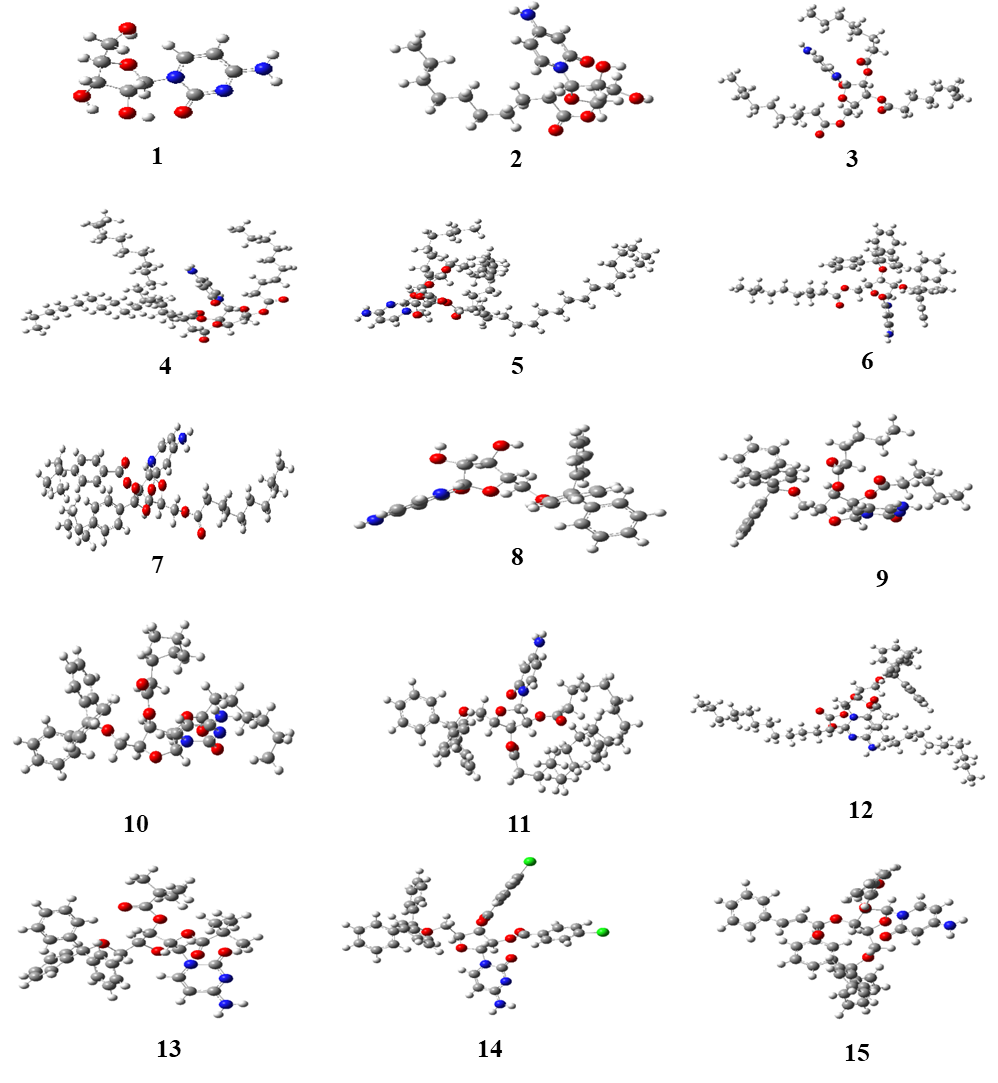


**Fig. S1.** **Optimized.** Structure of cytidine derivatives (**1-15**).


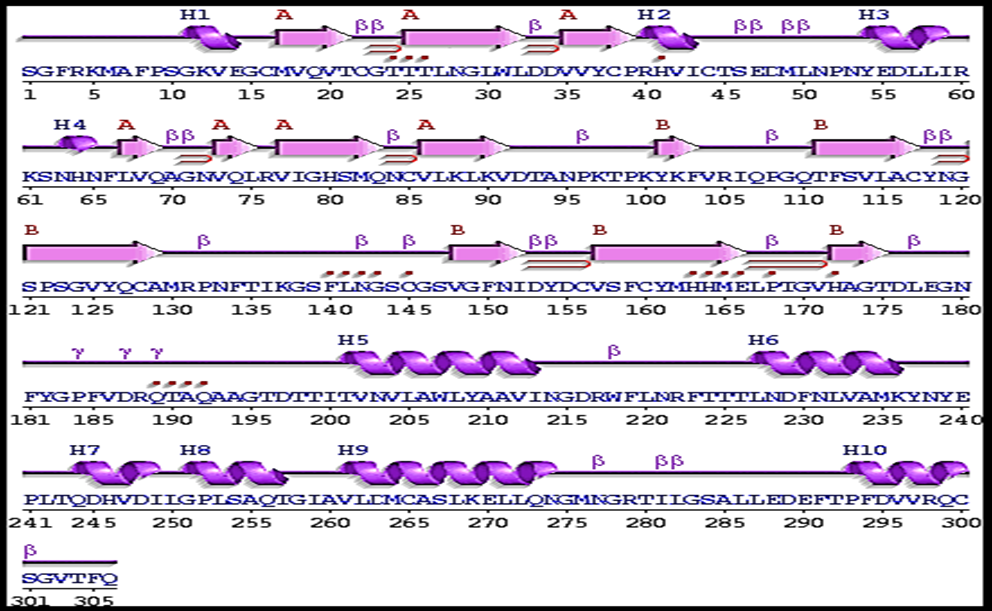


**Fig. S2. Multiple sequence alignment.** The closest homologs of RdRp (PDB: 6M71).

**Table S1. Structural views of the cytidine derivatives with SMILES.**

| Compounds | Molecular formula | Molecular weight | Compounds name | SMILES |
| --- | --- | --- | --- | --- |
| 1 | C_9_H_13_N_3_O_5_ | 242.22 | Cytidine | NC1=NC(=O)N(C=C1)C1OC(CO)C(O)C1O |
| 2 | C_19_H_31_N_3_O_6_ | 397.47 | 5´-*O*-Decanoylcytidine | CCCCCCCCCC(=O)OCC1OC(C(O)C1O)N1C=CC(N)=NC1=O |
| 3 | C_35_H_59_N_3_O_8_ | 649.87 | 5´-*O*-Decanoyl-2´,3´-di-*O*-octanoylcytidine | CCCCCCCCCC(=O)OCC1OC(C(OC(=O)CCCCCCC)C1OC(=O)CCCCCCC)N1C=CC(N)=NC1=O |
| 4 | C_51_H_91_N_3_O_8_ | 874.30 | 5´-*O*-Decanoyl-2´,3´-di-*O*-palmitoylcytidine | CCCCCCCCCCCCCCCC(=O)OC1C(COC(=O)CCCCCCCCC)OC(C1OC(=O)CCCCCCCCCCCCCCC)N1C=CC(N)=NC1=O |
| 5 | C_55_H_99_N_3_O_8_ | 929.86 | 5´-*O*-Decanoyl-2´,3´-di-*O*-stearoylcytidine | CCCCCCCCCCCCCCCCCC(=O)OC1C(COC(=O)CCCCCCCCC)OC(C1OC(=O)CCCCCCCCCCCCCCCCC)N1C=CC(N)=NC1=O |
| 6 | C_57_H_59_N_3_O_6_ | 882.11 | 5´-*O*-Decanoyl-2´,3´-di-*O*-(triphenylmethyl)cytidine | CCCCCCCCCC(=O)OCC1OC(C(OC(C2=CC=CC=C2)(C2=CC=CC=C2)C2=CC=CC=C2)C1OC(C1=CC=CC=C1)(C1=CC=CC=C1)C1=CC=CC=C1)N1C=CC(N)=NC1=O |
| 7 | C_41_H_55_N_3_O_8_ | 717.90 | 5´-*O-*Decanoyl-2´,3´-(4-*tert*-butylbenzoyl)cytidine | CCCCCCCCCC(=O)OCC1OC(C(OC(=O)C2=CC=C(C=C2)C(C)(C)C)C1OC(=O)C1=CC=C(C=C1)C(C)(C)C)N1C=CC(N)=NC1=O |
| 8 | C_28_H_27_N_3_O_5_ | 485.54 | 5´-*O*-(Triphenylmethyl)cytidine | NC1=NC(=O)N(C=C1)C1OC(COC(C2=CC=CC=C2)(C2=CC=CC=C2)C2=CC=CC=C2)C(O)C1O |
| 9 | C_40_H_47_N_3_O_7_ | 681.83 | 2´,3´-Di-*O*-hexanoyl-5´-*O*-(triphenylmethyl)cytidine | CCCCCC(=O)OC1C(COC(C2=CC=CC=C2)(C2=CC=CC=C2)C2=CC=CC=C2)OC(C1OC(=O)CCCCC)N1C=CC(N)=NC1=O |
| 10 | C_42_H_53_N_3_O_7_ | 709.87 | 2´, 3´-Di-*O*-heptanoyl-5´-*O*-(triphenylmethyl)cytidine | CCCCCCC(=O)OC1C(COC(C2=CC=CC=C2)(C2=CC=CC=C2)C2=CC=CC=C2)OC(C1OC(=O)CCCCCC)N1C=CC(N)=NC1=O |
| 11 | C_52_H_71_N_3_O_7_ | 850.15 | 2´,3´-Di-*O*-lauroyl-5´-*O*-(triphenylmethyl)cytidine | CCCCCCCCCCCC(=O)OC1C(COC(C2=CC=CC=C2)(C2=CC=CC=C2)C2=CC=CC=C2)OC(C1OC(=O)CCCCCCCCCCC)N1C=CC(N)=NC1=O |
| 12 | C_56_H_79_N_3_O_7_ | 906.26 | 2´,3´-Di-*O*-myristoyl-5´-*O*-(triphenylmethyl)cytidine | CCCCCCCCCCCCCC(=O)OC1C(COC(C2=CC=CC=C2)(C2=CC=CC=C2)C2=CC=CC=C2)OC(C1OC(=O)CCCCCCCCCCCCC)N1C=CC(N)=NC1=O |
| 13 | C_38_H_43_N_3_O_7_ | 653.77 | 2´,3´-Di-*O*-pivaloyl-5´-*O*-(triphenylmethyl)cytidine | CC(C)(C)C(=O)OC1C(COC(C2=CC=CC=C2)(C2=CC=CC=C2)C2=CC=CC=C2)OC(C1OC(=O)C(C)(C)C)N1C=CC(N)=NC1=O |
| 14 | C_42_H_33_N_3_O_7_Cl_2_ | 762.64 | 2´,3´-Di-*O*-(4-chlorobenzoyl)-5´-*O*-(triphenylmethyl) cytidine | NC1=NC(=O)N(C=C1)C1OC(COC(C2=CC=CC=C2)(C2=CC=CC=C2)C2=CC=CC=C2)C(OC(=O)C2=CC=C(Cl)C=C2)C1OC(=O)C1=CC=C(Cl)C=C1 |
| 15 | C_46_H_36_N_3_O_7_ | 742.80 | 2´,3´-Di-*O*-cinnamoyl-5´-*O*-(triphenylmethyl)cytidine | NC1=NC(=O)N(C=C1)C1OC(COC(C2=CC=CC=C2)(C2=CC=CC=C2)C2=CC=CC=C2)C(OC(=O)\C=C/C2=CC=CC=C2)C1OC(=O)\C=C/C1=CC=CC=C1 |

**Table S2**. **MMGBSA-based estimated binding free energies of complexes.**

| **RdRp-Compound 7** | | **RdRp-Compound 8** | | **RdRp-Compound 9** | | **RdRp-Compound 13** | | **RdRp-Compound 14** | |
| --- | --- | --- | --- | --- | --- | --- | --- | --- | --- |
| **Energy Component** | Average | Energy Component | Average | Energy Component | Average | Energy Component | Average | Energy Component | Average |
| **VDWALLS** | -52.10 | VDWALLS | -71.55 | VDWALLS | -58.64 | VDWALLS | -55.65 | VDWALLS | -60.38 |
| **EEL** | -37.40 | EEL | -35.64 | EEL | -30.00 | EEL | -33.51 | EEL | -34.61 |
| **EGB** | 17.10 | EGB | 22.65 | EGB | 25.64 | EGB | 20.32 | EGB | 25.11 |
| **ESURF** | -11.00 | ESURF | -10.17 | ESURF | -14.64 | ESURF | -10.14 | ESURF | -13.05 |
| **Delta G gas** | -89.5 | Delta G gas | -107.19 | Delta G gas | -89.62 | Delta G gas | -89.16 | Delta G gas | -94.99 |
| **Delta G solve** | 6.1 | Delta G solve | 12.48 | Delta G solve | 11 | Delta G solve | 10.18 | Delta G solve | 12.06 |
| **Total** | -83.4 | Total | -94.71 | Total | -78.62 | Total | -78.98 | Total | -82.93 |
